# Development of a country-specific CO_2_ emission factor for domestic anthracite in Korea, 2007–2009

**DOI:** 10.1007/s11356-012-0770-y

**Published:** 2012-02-04

**Authors:** Jeongwoo Lee, Jinsu Kim, Seungjin Kim, Gikyo Im, Seehyung Lee, Eui-Chan Jeon

**Affiliations:** 1 Department of Earth and Environmental Sciences, Sejong University, 98 Gunja-dong Gwangjin-gu, Seoul, 143-747 South Korea; 2 Cooperate Course for Climate Change, Sejong University, 98 Gunja-dong Gwangjin-gu, Seoul, 143-747 South Korea; 3 Department of Environment and Energy, Sejong University, 98 Gunja-dong Gwangjin-gu, Seoul, 143-747 South Korea

**Keywords:** Country-specific CO_2_, Emission, Domestic anthracite

## Abstract

**Introduction:**

Korea has been making efforts to reduce greenhouse gas (GHG) emissions, including a voluntary commitment to the target of a 30% reduction, based on business-as-usual of the total GHG emission volume, by 2020; 2006 IPCC Guidelines provided default values, applying country-specific emission factors was recommended when estimating national greenhouse gas emissions.

**Results and discussion:**

This study focused on anthracite produced in Korea in order to provide basic data for developing country-specific emission factor. This study has estimated CO_2_ emission factors to use worksheet of which five steps consisted according to the fuel analysis method.

**Conclusion:**

As a result, the average of net colorific value for 3 years (2007∼2009) was 4,519 kcal/kg, and the CO_2_ emission factor was calculated to be 111,446 kg/TJ, which is about 11.8% lower than the 2006 IPCC guidelines default value, and about 7.9% higher than the US EPA emission factor.

## Introduction

Korea has been actively making efforts to reduce greenhouse gas emissions, including a voluntary commitment to the target of a 30% reduction, based on business-as-usual (BAU) of the total greenhouse gas (GHG) emission volume, by 2020. In order to effectively reduce greenhouse gases and estimate potential reductions, it is necessary to identify and prepare an inventory of each source of gases and its related volume. Therefore, reliable data on greenhouse gas emission factors and active concentration is needed to prepare greenhouse gas inventories. Although 2006 IPCC Guidelines provided default values, applying country-specific emission factors was recommended when estimating national greenhouse gas emissions (IPCC, 2006). There have been ongoing studies on developing emission factors in many countries around the globe in order to estimate greenhouse gas emissions of combustion systems with the Tier 2 method, which consider the characteristics of national fuels (Quick and Glick, 2000; Sheng and Li, 2008). However, due to Korea’s lack of research on greenhouse gas emission factors, it inevitably relies on IPCC default values when writing national inventory reports.

Korea’s primary sources of energy in 2009 were: coal 68,604 toe; oil 102,336 toe; natural gas 33,908 toe; hydro power 1,213 toe; nuclear power 31,771 toe; and renewable energy 5,480 toe. Coal consumption accounted for about 28%, 108,378,000 t, a growth of 4,000,000 t compared with the previous year (Yearbook of Energy Statistics, 2010). Coal consumption is classified into anthracite and bituminous. Bituminous is wholly imported from abroad, while anthracite is both imported and produced domestically. Anthracite produced in Korea is a fossil fuel that contains 3∼7% of volatile matter, and 85∼87% fixed carbon.

In 2009, domestically produced anthracite accounted for 2,519,000 t, and was mainly used for power generation and civilian use. Anthracite consumption in 2009 was 3,309,000 t in total: 1,941,000 t (58.7%) for home and commercial use, 1,360,000 t (41.1%) for power generation, and 8,000 t (0.2%) for industrial use.

Although anthracite is mostly used in the energy sector and releases CO_2_ (Sibel Ozdogan, 1998), a lack of well-documented researches on emission factors of anthracite coal makes it difficult to set up a representative country-specific emission factor in Korea.

This study focused on anthracite produced in Korea in order to provide basic data for developing country-specific emission factors. In the process, carbon dioxide emission factors of domestic anthracite by the fuel analysis method outlined in the IPCC Guidelines were developed, using analysis data of domestic anthracite consumed by large consuming companies for 3 years, 2007–2009.

## Methods

### Anthracite sampling

A total of 190 anthracite samples, which are created in Permian of the Paleozoic era (Dai-Sung Lee, 1987), were collected in the Taebaek (35), Kyoungdong (38), Jangseong (37), Hwasun (33), Taean (21), and Seongha (26) areas from 2007 to 2009.

Methods for coal sampling are classified into mechanical sampling and manual sampling. This study used mechanical sampling, and collected samples using a sampling apparatus on a moving conveyor belt. A collected sample was then transported to the laboratory for processing, and manufactured into pulverized coal for fuel analysis. The analyzed sample went through several reductions and pulverizations, and eventually reduced to 250 μm, then preserved in a thermo-hygrostat on an air-dried basis (ASTM D 2234, 2004; ISO 1988, 1975). The assay values from different laboratories were compared by actual inspection of simultaneous analysis with KOLAS (Korea Laboratory Accreditation Scheme), in order to derive reliable data for analysis and for QA/QC.

### Anthracite analysis method

Fuel analysis is very important when estimating greenhouse gas emissions and emission factors (Roy et al. 2009; IPCC G/L 1996). International Organization for Standardization (ISO) and American Society for Testing Materials (ASTM) were mainly used, and Korea Standards (KS) were also partly used for fuel analysis. The proximate analysis and calorific values were gathered on an air-dried basis, and the ultimate analysis was on a dry basis, and those values were converted to as-received basis in order to estimate CO_2_ emission factors.

The colorimeter B was used to combust 1 g of sample to calculate its colorific value. This study used the IKA calorific value measurement apparatus (IKA-C2000, Germany) to analyze the colorific value of coal. This apparatus measures the gross colorific value of liquid and solid matter, and includes a water temperature controller (IKA-KV600, Germany), which maintains the temperature gap, and a bomb that burns the sample to generate heat. The colorific value was isoperibolic at 25°C mode, and the water temperature controller was set at 20°C (ISO 1928, 2009; ASTM D 2015-19, 1991; KS E 3707, 2006).

The ultimate analysis was conducted to analyze the carbon and hydrogen content of coal. The process heated and combusted a sample at about 800, absorbed the released carbon dioxide and vapor with an absorbent, measured their volume, and converted the carbon and hydrogen content into a percentage according to the total weight of the anhydrous sample. Also, this study used an automatic elemental analyzer (Thermo Finnigan-Flash EA 1112, USA) to oxidize the compound, based on the dynamic flash combustion method, separated it into a column, and analyzed it using TCD (ASTM D 3176-89, 2002; ASTM D 3178-89, 2002; KS E 3712, 1996).

In order to quantitate the fixed moisture, ash, volatile matter, and fixed carbon of coal, a proximate analysis was conducted using a thermo gravimetric analyzer (ELTRA Thermostep, Germany). It measured the reduced volume of moisture at 105°C, of volatile matter at 940°C with nitrogen injected, and the reduced volume of ash and fixed carbon at 750°C with oxygen injected. All of the above were measured three times at the relevant temperatures, and analyzed until the deviation was below 0.5 mg (ISO 17246, 2010; KS E 3705, 2003).

### Estimation of emission factor

The amount of greenhouse gas emissions is estimated by multiplication of fuel consumption by emission factors. This study has estimated CO_2_ emission factors of anthracite, using a five-step worksheet according to the fuel analysis method (Quick and Brill, 2002; IPCC, 1996) provided by the IPCC. Step 1 is to input data of carbon content, hydrogen content, and total quantity of moisture, and convert these into carbon content on an as-received basis. Step 2 is to convert the gross calorific value to the net calorific value, using the data derived from the calorific value analysis of fuel and ultimate analysis, and input fuel consumption. Step 3 is to calculate the amount of carbon emissions, considering carbon content (as-received basis), net calorific value, and oxidation quotient estimated from steps 1 and 2. The oxidation quotient of coal was presented as 0.98 on 1996 IPCC G/L, and was presented as 1 on 2006 IPCC G/L. Looking through the prior studies of domestic anthracite, the oxidation quotient can be applied as 1 (Seehyung Lee et al., 2011). Steps 4 and 5 are to divide the carbon emissions derived from step 3, by active concentration, calculate carbon emission factors per 1 TJ of fuel, and multiply the value by 44/12 to finally estimate CO_2_ emission factors (Eui-Chan Jeon et al., 2006).

## Results and discussion

### QA/QC

In order to estimate the reliability of the analysis data from laboratories, calorific value analysis, ultimate analysis, and proximate analysis from different laboratories were compared. Before comparing the analysis results, QA/QC was conducted first. In order to estimate reproducibility of the calorific value measurement apparatus, reference samples (manufacturer, IKA; calorific value, 6,320 ± 0.63 kcal/kg) were used. An electronic scale with an accuracy of 0.1 mg was used (Mettler Toledo-AB204S, Switzerland) to measure the weight of the samples. Table 1 presents the results of each reference sample repetitively analyzed five times in the same way. The average calorific value as a result of five times of repetitive analysis was 6,307 ± 4.27 kcal/kg, and the margin of error with the calorific value of reference samples was about 9 kcal/kg. It was evaluated to have very excellent reproducibility as the relative standard deviation among samples was 0.07%.

**Table 1:** Results of reproducibility test for calorific value

Time	Mass of standard (g)	Gross calorific value (kcal/kg)
1	0.5327	6,307
2	0.5342	6,311
3	0.5333	6,312
4	0.5327	6,303
5	0.5324	6,303
	Mean	6,307
	SD	4.27
	RSD (%)	0.07

BBOT (2,5-bis(5-*tert*-butyl-benzoxazolyl)thiophene) reference sample was used to evaluate reproducibility of the elemental analyzer; 1.5∼2.0 mg of a sample was injected and measured with a precision balance (Mettler Toledo-MX5, Switzerland) which could measure to a minimum of 0.001 mg. After preprocessing of the sample, it was injected into an auto sampler to be analyzed. For ultimate analysis, a 2-m long column (ParaQX) was used. And the flux was set as: carrier gas 140 mL/min, oxygen 240 mL/min, and reference gas 100 mL/min. The furnace was set at 900°C, the oven at 70°C, and the result of three times of repetitive analysis is shown in Table 2. The absolute difference was 0.19∼0.33% for carbon and 0.03∼0.06% for hydrogen.

**Table 2:** Results of reproducibility test for elemental analysis

Time	Sample type	Injection weight (mg)	Element content (%)	
			C	H
1	BBOT	1.882	72.53	6.09
	Unknown	1.857	72.72	6.14
	Absolute difference (%)		0.19	0.05
2	BBOT	1.823	72.53	6.09
	Unknown	1.848	72.81	6.15
	Absolute difference (%)		0.28	0.06
3	BBOT	1.868	72.53	6.09
	Unknown	1.724	72.86	6.12
	Absolute difference (%)		0.33	0.03

Proximate analysis had a quality control experiment through the analysis result: 0.75% sulfur coal PROX-X, 3% sulfur coal PROX-X, 5% sulfur coal PROX-X (IARM HC30075C, IARM HC30300B, IARM HC30500B, dry basis analysis). The standard deviation (SD) of each item according to the analysis results of reference samples is shown in Table 3: moisture 0.03∼0.05%, volatile matter 0.05∼0.14%, ash 0.002∼0.04%, and fixed carbon 0.09∼0.15%, which showed excellent reproducibility.

**Table 3:** Results of reproducibility test for proximate analysis

Sample type	Time	Weight (mg)	Element content (%)			
			W	VM	A	FC
0.75% sulfur coal PROX-X	1	954.0	2.24	34.13	6.95	56.68
	2	1,013.9	2.31	34.17	6.95	56.57
	3	959.7	2.23	34.07	6.95	56.76
		Mean	2.26	34.12	6.95	56.67
		SD	0.04	0.05	0.00	0.09
		RSD (%)	1.85	0.15	0.03	0.16
3% sulfur coal PROX-X	1	953.5	1.45	35.48	8.47	54.60
	2	950.1	1.43	35.76	8.40	54.40
	3	962.9	1.53	35.59	8.43	54.45
		Mean	1.47	35.61	8.44	54.48
		SD	0.05	0.14	0.03	0.10
		RSD (%)	3.47	0.40	0.39	0.19
5% sulfur coal PROX-X	1	964.2	1.52	30.30	21.14	47.04
	2	971.9	1.55	30.33	21.11	47.00
	3	964.3	1.59	30.07	21.06	47.29
		Mean	1.55	30.23	21.10	47.11
		SD	0.03	0.14	0.04	0.15
		RSD (%)	1.99	0.47	0.18	0.32

### Mott-Spooner test

We used the Mott-Spooner to verify the analyzed values of the anthracite. This study concluded that. So, the data in this range is considered to be acceptable (Peter H. Given et al., 1986).

This study analyzed the difference between the laboratory value and the value calculated from the Mott-Spooner test (Table 4). According to former study conducted, the data is considered to be acceptable when the difference between the calculated caloric and laboratory analyzed result value is ±140 kcal/kg. In this study, the result showed that the Mott-Spooner difference is −333∼135 kcal/kg, and most samples were within the range of ±140 kcal/kg, which is generally allowed. However, some samples exceed the range, because coal found in Korea is considered to be of a lower rank (Peter H. Given et al., 1986).

**Table 4:** The comparison with calculated and analyzed calorific value

Sample number	C	H	O	N	S	Dry kcal/kg	Dry kcal/kg calculated	Mott-Spooner difference
A-1	60.30	0.13	0.36	0.05	0.69	4,906	4,888	18
A-2	62.45	0.86	0.22	0.28	0.51	5,311	5,309	2
A-3	62.45	0.86	0.22	0.28	0.51	5,226	5,309	−83
A-4	63.80	0.63	0.62	0.26	0.41	5,305	5,323	−18
A-5	63.80	0.63	0.62	0.26	0.41	5,276	5,323	−47
A-6	63.83	0.94	1.66	0.33	0.53	5,505	5,397	108
A-7	63.83	0.94	1.66	0.33	0.53	5,327	5,397	−70
B-1	61.13	0.90	0.39	0.40	0.76	5,250	5,216	34
B-2	61.13	0.90	0.39	0.40	0.76	5,063	5,216	−153
B-3	63.63	1.08	2.00	0.69	0.77	5,303	5,422	−119
B-4	61.13	0.90	0.39	0.40	0.76	5,326	5,216	110
B-5	61.10	0.50	0.91	0.43	0.59	4,962	5,056	−94
B-6	61.10	0.50	0.91	0.43	0.59	4,970	5,056	−86
B-7	63.10	0.50	0.48	0.48	0.58	5,055	5,232	−177
C-1	58.45	0.91	1.38	0.38	0.46	4,923	4,963	−40
C-2	58.45	0.91	1.38	0.38	0.46	4,951	4,963	−12
C-3	58.45	0.91	1.38	0.38	0.46	4,897	4,963	−66
C-4	59.37	1.04	2.63	0.33	0.34	4,950	5,035	−85
C-5	58.97	1.25	0.88	0.40	0.43	4,889	5,137	−248
C-6	57.85	1.28	1.54	0.10	0.46	4,963	5,035	−72
C-7	61.35	0.80	0.20	0.42	0.45	4,999	5,200	−201
D-1	61.83	0.75	1.86	0.21	0.11	5,043	5,156	−113
D-2	61.83	0.75	1.86	0.21	0.11	5,047	5,156	−109
D-3	61.83	0.75	1.86	0.21	0.11	4,959	5,156	−197
D-4	61.50	0.89	3.72	0.21	0.11	4,855	5,112	−257
D-5	60.07	0.98	1.80	0.17	0.26	4,765	5,098	−333
D-6	55.10	1.11	2.76	0.10	0.18	4,565	4,708	−143
D-7	62.85	0.18	0.88	0.22	0.28	4,899	5,082	−183
E-1	58.87	0.98	2.97	0.33	0.75	4,851	4,972	−121
E-2	59.07	1.17	1.10	0.16	0.58	5,092	5,114	−22
E-3	59.07	1.17	1.10	0.16	0.58	4,878	5,114	−236
E-4	58.23	0.94	1.81	0.35	0.43	5,075	4,940	135
E-5	58.23	0.94	1.81	0.35	0.43	4,938	4,940	−2
E-6	58.23	0.94	1.81	0.35	0.43	4,664	4,940	−276
E-7	58.87	0.98	2.97	0.33	0.75	4,815	4,972	−157
F-1	76.67	0.62	1.37	0.10	0.03	6,386	6,318	68
F-2	79.00	0.32	0.34	0.10	0.02	6,351	6,439	−88
F-3	78.00	0.83	0.60	0.10	0.01	6,415	6,523	−108
F-4	79.10	0.33	0.34	0.10	0.00	6,251	6,450	−199
F-5	76.50	0.74	1.24	0.10	0.01	6,285	6,349	−64
F-6	76.50	0.74	1.24	0.10	0.01	6,273	6,349	−76
F-7	76.50	0.74	1.24	0.10	0.01	6,369	6,349	20

### Comparative analysis by laboratories

This study sent part of a collected sample to KOLAS and conducted comparative analysis of the same sample in order to evaluate reliability of the analysis data of domestic anthracite. Permissible tolerance of each item is, as shown in Table 5, presented at “ASTM D7582”, “ASTM D5373”, and “KS E 3709”: errors below, calorific value 40 kcal/kg, carbon 1.5%, nitrogen 0.7%, volatile matter 1.40%, and ash 0.80% are permissible.

**Table 5:** Tolerance of analysis in laboratory

Category	Caloric value	C	H	VM	A
	kcal/kg	%	%	%	%
Tolerance of interior	40	0.4	0.18	0.40	0.40
Tolerance of interloculus	80	1.5	0.70	1.40	0.80

ASTM D5373-02, 2007; ASTM D7582-10, 2010; KS E 3709, 2009

Comparison of the error of each item based on the analysis results from different laboratories, as shown in Table 6, is as follows: calorific value 13∼58 kcal/kg, carbon 0.13∼1.01%, nitrogen 0.03∼0.42%, volatile matter 0.16∼0.70%, and ash 0.09∼0.64%; results were shown to be excellent as the errors among laboratories were within the permissible tolerance as presented in Table 5.

**Table 6:** Result of analysis of anthracite in Korea in 2009

Time	Institution	Caloric value	C	H	M	VM	A	FC
		kcal/kg	%	%	%	%	%	%
1	Laboratory	4,618	64.16	0.7	2.08	5.83	31.83	60.26
	KOLAS	4,637	64.03	0.73	2.82	5.99	32.03	59.16
	Error	19	0.13	0.03	0.74	0.16	0.20	1.10
2	Laboratory	4,595	64.06	0.72	4.13	5.91	32.94	57.02
	KOLAS	4,582	63.05	1.14	4.41	5.21	33.09	57.29
	Error	13	1.01	0.42	0.28	0.70	0.15	0.27
3	Laboratory	4,608	63.48	0.98	3.7	6.6	32.99	56.71
	KOLAS	4,666	63.25	1.1	4.08	6.42	32.35	57.15
	Error	58	0.23	0.12	0.38	0.18	0.64	0.44
4	Laboratory	4,566	62.2	1.05	3.96	5.99	33.57	56.48
	KOLAS	4,537	61.62	1.15	3.81	6.38	33.66	56.15
	Error	29	0.58	0.10	0.15	0.39	0.09	0.33
5	Laboratory	4,572	63.1	0.8	4.58	5.17	32.69	57.56
	KOLAS	4,624	63.29	0.83	3.65	5.74	33.14	57.47
	Error	52	0.19	0.03	0.93	0.57	0.45	0.09

*C* carbon, *H* hydrogen, *M* moisture, *VM* volatile matter, *A* ash, *FC* fixed carbon

### Analysis results of anthracite

The results of ultimate analysis and the calorific value of anthracite, which was domestically produced in 2007–2009, are presented in Table 7. The carbon content of domestic anthracite was 62.42% in 2007, 63.27% in 2008, and 63.46% in 2009; the average was 62.98%. Nitrogen content was 1.05% in 2007, 1.07% in 2008, and 0.79% in 2009; the average was 0.99%. And its calorific value was 4,542 ± 127 kcal/kg in 2007, 4,515 ± 103 kcal/kg in 2008, and 4,487 ± 101 kcal/kg in 2009; the average was 4,519 ± 112 kcal/kg.

**Table 7:** Annual data of calorific value and C and H of anthracite produced in Korea

Year	Output	Gross calorific value (air dried)	C (air dried)	H (dried)
	Thousand tons	kcal/kg	%	%
2007	2,886	4,542 ± 127	62.42	1.05
2008	2,773	4,515 ± 103	63.27	1.07
2009	2,519	4,487 ± 101	63.46	0.79
Average		4,519 ± 112	62.98	0.99

### CO_2_ emission factor of anthracite

The CO_2_ emission factors from this study and from other institutions are presented in Table 8. The CO_2_ emission factor calculated from this study is 111,446 kg/TJ, which is about 11.8% higher than the IPCC default value (98,300 kg/TJ), and about 7.9% higher than the US EPA emission factor (102,632 kg/TJ). The amount of anthracite consumed in 2009 was 3,309,000 t, and the volume of CO_2_ emissions was about 6,977,000 t_,_ according to the emission factor calculated from this study. It is calculated to be 6,154,000 t of CO_2_ based on the IPCC default value, and 6,426,000 t of CO_2_ based on the US EPA emission factor.

**Table 8:** Compare C and CO_2_ emission factors of institutional anthracite

Category	C emission factor	CO_2_ emission factor
	(kg/GJ)	(kg/TJ)
This study (as-received basis, 2011)	30.39	111,446
Previous study^a^ (as-received basis, 2006)	30.45	111,650
IPCC^b^	26.8	98,300
US EPA^c^	28.0^d^	102,632

^a^Greenhouse gas emission factor development for coal-fired power plants in Korea (2010)

^b^2006 IPCC Guidelines volume 2 energy

^c^1999 US average of coal electric power plant (NCV is about 5% less than the GCV_2006 IPCC G/L)

^d^Conversion from CO_2_ emission factor

Comparing the CO_2_ emission factor (111,446 kg/TJ) with prior studies, it is about 0.2% lower than the value of 111,650 kg/TJ calculated by Eui-Chan Jeon et al. (2010). There was not a big change in the emission factors of domestic anthracite, but Table 9 shows that the calorific value and carbon content was measured to be about 3% lower. Based on these results, we can see that the calorific value of domestic anthracite is gradually decreasing annually.

**Table 9:** Trend of calorific value of anthracite produced in Korea

Category	C as-received base	Calorific value	Carbon emission factor
	%	kcal/kg	Ton C/TJ
2002∼2004 average^a^	58.20	4,565 ± 309	30.45
2007∼2009 average	57.51	4,519 ± 112	30.39

^a^Greenhouse gas emission factor development for coal-fired power plants in Korea (2010)

## Conclusions

This study focused on domestically produced anthracite and estimated its greenhouse gas emissions and emission factors. When estimating CO_2_ emission factors, it followed the fuel analysis method from the IPCC G/L. As a result of fuel analysis, the average net colorific value of domestic anthracite for three recent years (2007–2009) was calculated to be 4,519 kcal/kg, which is 46 kg/kcal lower than the prior research result of 4,565 kg/kcal (2002–2004). The CO_2_ emission factor was calculated to be 111,446 kg/TJ, which is about 11.8% lower than the IPCC default value, and about 7.9% higher than the US EPA emission factor. Using the CO_2_ emission factor from this study, the emission volume can be calculated to be approximately 6,977,000 t.

The number of mines in Korea, along with the supply of coal, is decreasing. Anthracite for civilian use will continue at the present rate due to the Coal Industry Act, but anthracite for power generation is anticipated to gradually become less available. As a result, power plants that use anthracite started to import their coal from North Korea or Vietnam in 2008. Although the weight of imported anthracite is low today, it is anticipated to slowly increase due to the present situation. Consequently, the calorific value, the value of carbon, and the inherent moisture will all be changing according to the composition of the imported anthracite, and this will necessarily affect the amount of greenhouse gas emissions. Therefore, studies should continually be conducted on a regular basis to ensure reliable estimates of country-specific emission factors.
